# Incorporating risk preferences of patients in the valuation of immune checkpoint inhibitors for non-small cell lung cancer

**DOI:** 10.3389/fonc.2023.1027659

**Published:** 2023-03-08

**Authors:** Remziye Zaim, W. Ken Redekop, Carin A. Uyl-de Groot

**Affiliations:** Erasmus School of Health Policy & Management, Erasmus University, Rotterdam, Netherlands

**Keywords:** immune checkpoint inhibitor, non-small cell lung cancer, value assessment, risk preferences, hope

## Abstract

Immunotherapy offers a distinctive mechanism of action compared to traditional treatments, arising from additional value dimensions that may not be captured in standard health technology assessments. Cancer patients may have the expectation that immunotherapy provides durable, long-term survival gains. Moreover, some patients may be willing to take a ‘risk’ to undergo immunotherapy to achieve better survival outcomes. We reviewed quantitative methods that explored patients’ risk preferences in their non-small cell lung cancer (NSCLC) treatment choices, in PubMed (MEDLINE), from January 1, 2015, until July 1, 2022. The consideration of a value dimension (‘hope’) based on patients’ risk-seeking preferences is specifically addressed for the valuation of immune checkpoint inhibitors in NSCLC. We reported that the quantitative methods that aim to measure patients’ risk preferences or ‘hope’ empirically are emerging. Value assessments should not only comprise survival improvements for the mean or median patient but also consider methods that reflect durable, long-term overall survival gains for risk-seeking patients. However, the published evidence for incorporating ‘hope’ based on patients’ stated preferences for uncertain treatment profiles is not strong, and future research could strengthen this evidence base. We encourage further research on the development and validation of quantification methods to incorporate ‘hope’ and risk preferences of patients treated with immunotherapy for NSCLC and beyond.

## 1 Introduction

Immunotherapy represents a significant breakthrough in the treatment of cancer. Immune checkpoint blockade is an effective therapeutic strategy that harnesses the immune system to generate an antitumor response (1, 2). Immune checkpoint inhibitors (ICIs) targeting cytotoxic T lymphocyte-associated protein 4 (CTLA-4) and programmed cell death protein 1/programmed cell death ligand 1 (PD-1/PD-L1) have been integrated into the standard of care for patients with various cancers, including non-small cell lung cancer (NSCLC) (2). To explore the clinical efficacy and safety of these ICIs, thousands of clinical trials are underway (3). Clinical trial guidelines promote transparent and accurate reporting of patient-reported outcomes (PROs), in an effort to facilitate the interpretation and limitations of complex patient data (4, 5). Also, there is evidence that monitoring treatment side effects in real-time can improve outcomes for patients with cancer, including a potential benefit in survival rates (2, 6). However, patients may express their preferences for innovative durable therapies (e.g., immune checkpoint inhibitors) with uncertain levels of benefit, with a likelihood of a good outcome (7). Moreover, some patients (i.e., risk-seeking patients) may be willing to take additional risks (at end of life situations) to increase the probability of a survival outcome. This preference could be attributed to the evidence that although individuals may in general be risk averse, in situations where they face very poor prospects, they may become risk-seeking (8–10).

Recently, there have been theoretical research efforts to consider additional dimensions of benefit, including patients’ risk preferences in their treatment choices (11). Value assessment frameworks quantifying clinical and economic outcomes of health technologies are often used to quantify the net value of NSCLC therapies (12). For example, the value frameworks used by the National Institute for Health and Care Excellence (NICE) and the Institute for Clinical and Economic Review (ICER) use average health-related quality of life (HRQoL) as a key measure of health benefit (13–15). Similarly, the value framework of the European Society for Medical Oncology enables optional weighting of treatment outcomes based on HRQoL (16). Moreover, the value framework of the American Society of Clinical Oncology (ASCO) recognizes the value of additional survival gains as part of their evaluation process (17).

Quality-adjusted life year (QALY), as a health benefit measure, is based on the assumption that marginal utility equals average utility in both quality of life and life year, and that the utility is linear, not concave, or equivalently that patients are risk-neutral (9). Introduction of risk preferences may provide a way to incorporate variability in health benefits that have not been incorporated in standard value assessments. To address the potential impact of incorporating patients’ risk-seeking preferences in value assessment frameworks, we reviewed the evidence on quantitative methods and reflected upon their potential incorporation in the valuation of ICIs in NSCLC. A consideration of ‘hope’ as a value dimension based on patients’ risk-seeking preferences is specifically addressed.

## 2 Identification of valuation methods for patients’ risk preferences

We searched quantitative methods that explored the risk preferences of patients in their cancer treatment choices, in PubMed (MEDLINE), from January 1, 2015, until July 1, 2022. (see **Supplementary Table 1**) The consideration of a value dimension (‘hope’) based on patients’ risk-seeking preferences is specifically addressed for the valuation of ICIs in NSCLC. Study findings were summarized descriptively.

## 3 Consideration of risk preferences in standard value assessment

We explored standard value assessment foundations and investigated methods specifically for patients’ risk preferences in their treatment choices. Although the standard value assessment frameworks provide a useful starting point, in the absence of broader value considerations, some limitations may occur. These limitations could potentially lead to suboptimal resource allocation decisions, such as distorted signals to innovators, and imprecise evaluation of durable medical technologies (i.e., ICIs) (11). Although there are strengths of the standard value assessments that use the ‘incremental cost-effectiveness ratio’ methodology, a number of limitations exist as well. In the standard value assessments, average health benefits and costs are included in the valuation of technologies. However, patient preferences and clinical practice may differ from such average outcomes. While HTA agencies use a number of criteria to make coverage decisions, institutions such as the NICE in the United Kingdom focus on cost-effectiveness and affordability as key determinants of their appraisal decisions (18). Although healthcare budgets are limited in such jurisdictions, there is a strong desire to reimburse innovative cancer therapies with some uncertainty, as demonstrated by the Cancer Drug Fund of the National Health Services in England (19). Similarly, Canada has a distinct review process for reimbursement of oncology drugs. The Canadian Agency for Drugs and Technologies in Health pan-Canadian Oncology Drug Review is responsible for the assessment of cancer treatments (20). The methods used by these agencies, however, do not explicitly capture patients’ risk preferences or incorporate ‘hope’ for risk-seeking patients.

### 3.1 “Hope” for risk-seeking patients

**Figure 1:**
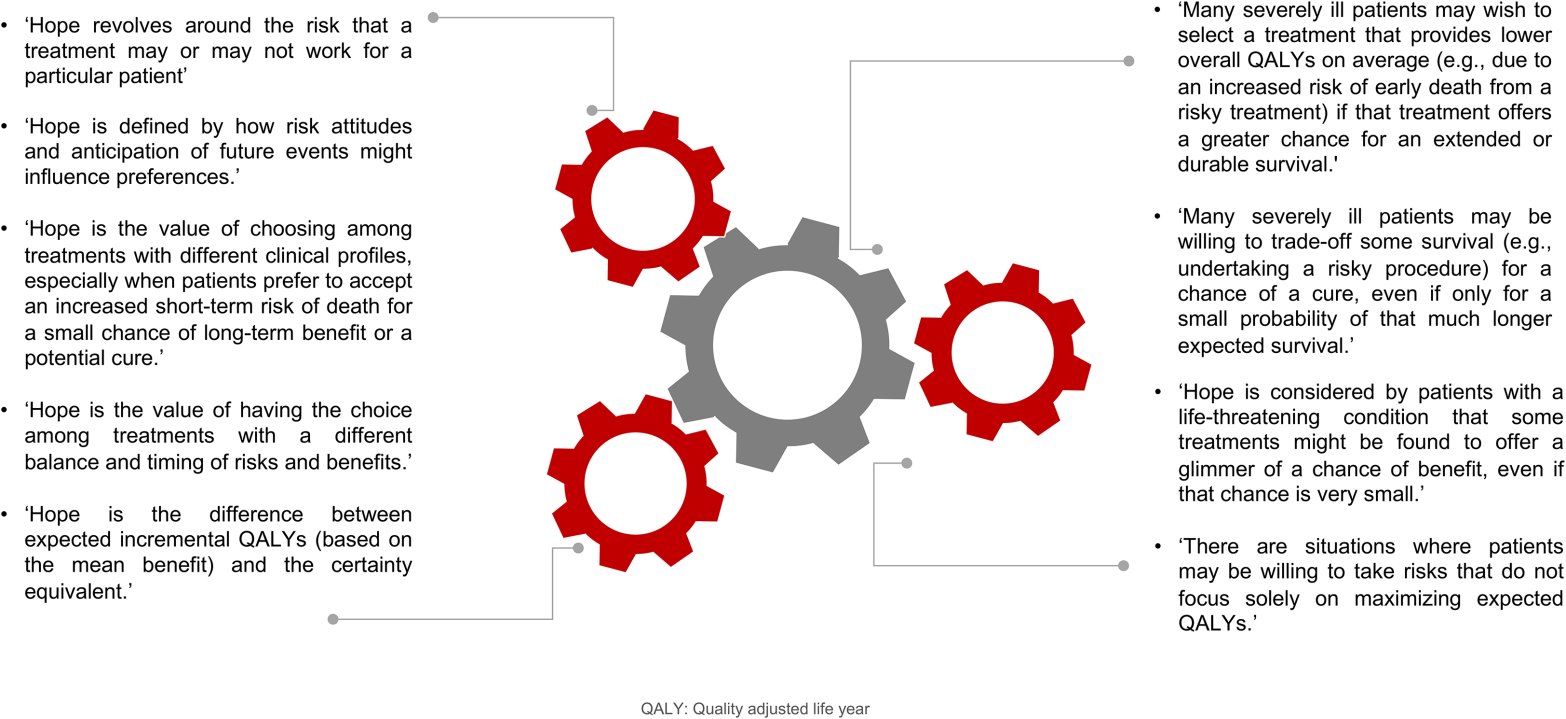
Definitions of ‘hope’ for risk-seeking patients. QALY, Quality adjusted life year.

The consideration of ‘hope’ as a value dimension based on patients’ risk-seeking preferences is becoming evident for the valuation of therapies in NSCLC. However, we found that there are a number of definitions that may include ‘hope’ in the valuation of health technologies in the context of risk. **Figure 1** shows multiple definitions of ‘hope’ identified in this context. Regardless of its nomenclature, the value of choosing among treatments with different clinical profiles, especially when some patients are willing to take the risk for a small chance of durable survival benefit or a potential cure, creates conditions for further research.

### 3.2 Valuation of risk preferences in patients treated with ICIs for NSCLC

To explore the risk preferences of patients for durable overall survival benefits, Shafrin et al., 2017 prospectively surveyed lung and melanoma cancer patients and their physicians (21). For the purposes of our study, however, we solely described methods and findings pertaining to lung cancer. The authors specifically compared physicians’ view of a chance of durable survival (at the tail of the survival curve), independent of average survival, to that of the patients. The results of this survey were used to determine “how patients and their physicians value therapies that offer a likelihood of durable survival outcomes” (21). “Durable survival treatments were calibrated based on survival outcomes (i.e., 66 months of follow-up) from the pivotal trials of nivolumab investigated in patients with advanced NSCLC” (18). The primary end-point was “the proportion of respondents who selected a therapy with a variable survival profile, (with some patients experiencing long-term durable survival and others experiencing shorter survival), compared to a therapy with a fixed survival duration” (21). Fixed survival was hypothetical, where “all patients were assumed to live for a specified period of time prior to their death” (21). Parameter estimation by sequential testing (“PEST”) was applied to “calculate to estimate the duration of survival that would make patients or physicians indifferent between fixed survival and therapy with durable survival.” (21) “PEST is an adaptive elicitation technique that determines the stimulus value for each new question using responses to the previous question.” (21, 22) In the study, patients, and physicians continued to receive questions until an indifference point was reached, or until 10 questions were answered (21).

Overall, the analysis comprised 84 lung cancer patients and 96 physicians (21). There were two primary endpoints: “1) whether the respondent preferred a durable survival therapy compared with a fixed survival therapy”; and “2) the indifference point in terms of survival between a durable survival therapy and a fixed survival therapy.” (21) For lung cancer, “65.5% of patients preferred the therapy with a variable survival profile, compared with 40.8% of physicians (Δ=24.7%; P 0.001).” (21) “Patients’ indifference point indicated that therapies with a variable survival profile were preferred unless the treatment with fixed survival had 11.6 months longer mean survival” (21). “Physicians were prescribing treatments with a fixed survival if the treatment had 1.0 months shorter survival compared to the uncertain survival profile” (21). Based on risk preference distributions of the Kaplan-Meier curve estimations, Shafrin et al. assumed a constant relative risk aversion utility function (23). To compare the indifference point with the certainty equivalent, t-tests were performed. Patients’ indifference point among lung cancer therapies with durable and fixed survival was “41.6 months (i.e., 11.6 months greater than the average survival at 30 months)” (21). In contrast, the physicians’ indifference point was 29 months (21). The overall “indifference point was 12.6 months greater (P < 0.001) for patients compared to the physicians.” (21) Applying a constant relative risk aversion (RRA) utility function, the authors estimated that “patients are risk-seeking (RRA = +0.39 for NSCLC; P < 0.001), and physicians are risk neutral for lung cancer treatments (RRA = –0.03; P = 0.523).” (21) “The patient’s utility function was u(x) = x^1.39^ and the physician’s utility function was u(x) = x^0.97^” (21).

Shafrin et al. showed that “lung cancer patients were willing to give up 38.7% of an average survival for a likelihood of durable survival” (21). The patient preferences reported in this study are consistent with the prospect theory, which predicts that people may be risk-seeking in circumstances starting below their reference point (24, 25). However, it should be noted that discordant preferences do not necessarily mean that “physicians override their patients’ desires” (26). Some patients may prefer that their physicians make treatment decisions on their behalf (27). Nonetheless, physicians’ decisions may not always be aligned with that of patients’ interests (28). Therefore, it is important to conduct additional research to understand ‘when, why, and how’ physicians may implicitly or explicitly substitute their perspectives on patients’ behalf.

Shafrin et al., 2018 examined whether incorporating additional value considerations was influential on the cost-effectiveness estimates (26). Previous research suggested that broader societal benefits could be considered in value assessments (29, 30). For example, assessments could potentially include patients’ treatment preferences that offer durable survival benefits, instead of average outcomes (21, 31). Building upon a previously published cost-effectiveness analysis (32), Shafrin et al. studied patients in Canada with advanced squamous NSCLC treated with second-line nivolumab. The authors used the net monetary benefit framework (33), to calculate cost-effectiveness estimates (34). The authors conducted their analyses from three perspectives; namely the “traditional payer”, “traditional societal” and “broad societal” perspectives (34). The traditional payer perspective was built based on a model developed in Canada by the Goeree et al. (32) Goeree et al. extrapolated progression-free survival (PFS) and overall survival (OS) Kaplan Meier curves from the CheckMate 017 Phase 3 clinical trial (35). Goeree et al. estimated the proportion of patients in “progression-free, progressed disease and death” health states, for a time horizon of 10 years (32). The CheckMate 017 trial investigated the clinical and safety outcomes of “nivolumab (3 mg/kg every two weeks) compared to docetaxel (75 mg/m^2^ every three weeks) for previously treated patients with advanced squamous NSCLC” (35). The results of this trial showed “significant improvements in median OS and PFS for nivolumab patients compared to docetaxel patients (OS: 9.2 vs. 6.0 months, HR: 0.59; PFS: 3.5 months vs. 2.8 months, HR: 0.62).” (35) Goeree et al. reported that based on the CheckMate 017 clinical trial results, the incremental cost-effectiveness ratio (ICER) was “ Canadian dollars (CA)$151,560 per QALY gained” (32).

To explore a broader societal perspective, Shafrin et al. quantified ‘hope’ in addition to caregiver burden, insurance value, and option value of the nivolumab treatment in patients with advanced NSCLC. For the purposes of our review, however, we focused our descriptive summary on the valuation of ‘hope’ or patients’ risk preferences. The Checkmate 017 clinical trial showed that the “two-year overall survival for nivolumab was 24%, and reduced to 16% at five years” (35). To quantify ‘hope’ (31) the authors first measured the difference in the expected survival between nivolumab and docetaxel using the Kaplan-Meier curve obtained from Goeree et al. (32) Subsequently, they estimated the “certainty equivalent between the two treatments using a utility function based on the previously reported risk aversion estimates for NSCLC patients” (21). The authors assumed that the difference between the expected survival difference and this estimated certainty equivalent provides a valuation method to quantify ‘hope’. Using this method, Shafrin et al. estimated an “additional QALY gain of 0.039, beyond the baseline estimate of 0.66 QALYs” (32), at an additional cost of CA$ 5,850 (26). The ICERs for nivolumab compared to docetaxel were “$151,560, $141,344, and CA$80,645 per QALY gained from the traditional payer, traditional societal, and broader societal perspectives, respectively” (26).

Similarly, the researchers at the Innovation and Value Initiative (IVI) in the US developed a patient-centered value assessment, which incorporated ‘hope’ into a cost-effectiveness model for advanced NSCLC (36). In this model, treatment regimens for the study population comprised ICIs for the progressed disease after second-line therapy. From a clinical perspective, this model requires an update based on the modifications in the latest NSCLC guidelines. From an economic perspective, in the IVI’s four-state model, patients were assumed to begin first-line (1L) treatment in stable disease (S1) and can either experience disease progression and consequently transition to second-line (2L) treatment (P1/S2), or death (D) (36). With 2L treatment, patients can either experience disease progression (P2) or death (D). At P2, patients begin 2L+ treatment (including ICIs) and remain in this state until death (36). IVI’s NSCLC model specifically focused on the quantification of ‘hope’ to address patients’ risk attitudes when “treatments with equivalent expected health benefits differ in their overall benefit distributions” (36). This is assumed to be true when a benefit distribution has a longer-term survival for some patients. The authors of this study quantified ‘hope’ as the “difference between expected incremental QALYs (based on the mean benefit) and the certainty equivalent” (36). The certainty equivalent was then defined as “the number of QALYs that a patient would need to obtain to be indifferent between the comparator and comparative treatment strategy, with alternative distribution of survival outcomes” (21, 36). This definition was again based on the Shafrin et al. study which showed that “patients place a high value on treatments with a higher probability of durable overall survival benefits” (21).

Although these NSCLC case studies illustrate potential methods to quantify patients’ risk preferences or ‘hope’ when treated with an ICI, there are a number of methodological and practical issues that require careful consideration. Standard value assessments including cost-effectiveness assume risk neutrality (37). Ignoring risk preferences may underestimate or overestimate the value of interventions for different indications (9, 10). Consideration of two competing interventions with the same average survival, one with greater uncertainty, would be deemed comparable in a conventional or standard cost-effectiveness assessment. For some patients, however, this uncertainty may indicate that they have a treatment preference. As quantified by Shafrin et al. (21), the certainty equivalent may comprise health benefits (i.e., QALYs) that a patient may be indifferent among ICIs or the alternative treatment strategy. Estimations of ‘hope’ may indicate that the distribution of health benefits should not only be characterized by their variance but also by their skewness. For risk-seeking patients, including those with NSCLC, some may prefer the treatment option with durable survival, at the expense of a higher risk of dying earlier (31). In the Shafrin et al. case study (26), there were data limitations specific to nivolumab or NSCLC to measure broader societal benefits. Even if such data were available, it may not be possible that the studies would cover all indications at the time of value assessments (26). Moreover, after clinical trial results are published, there is significant uncertainty related to the long-term benefits of ICIs, owing to the relatively short follow-up period of most immunotherapy clinical trials. Therefore, payers and the HTA agencies may under or overestimate treatment value, extrapolate value estimates from different studies, indications, and conditions, or consider alternative assumptions. Nonetheless, these case studies illustrate that quantification of patients’ risk preferences, referred to as ‘hope’ is a research area that requires further investigation and validation in NSCLC patients, specifically for those treated with an ICI.

### 3.3 Valuation of patients’ risk preferences beyond immunotherapy in NSCLC

Studies included in our review also highlighted potential ways to characterize patients’ risk preferences (“hope”) beyond NSCLC. Valuation of patients’ risk preferences or ‘hope’ in the assessments of health technologies has yet to gain traction, in part due to reliance on established practices, and the lack of motivations to improve existing frameworks (38). An alternative viewpoint is that the use of standard value assessment methods is sufficient, because “cost-effectiveness estimates are only an input to, and not a substitute for, a deliberative decision-making process that allows for additional elements of value to be contextualized into the process without the need for formal quantification” (39). Regardless of the viewpoints, patients’ risk preferences, their relative importance for some indications, valuation methods, and potential quantification methods remain areas for further research. In this section, we highlight key studies that provide distinct methods to help quantify ‘hope’ and incorporate patients’ risk preferences beyond the treatment of NSCLC.

Lakdawalla et al., estimated patients’ risk preferences in melanoma, breast cancer, and other solid tumor patients (31). The authors recommended incorporating ‘hope” into the valuation of end-of-life treatments or considering a higher cost-effectiveness threshold for treatments at the end of life. The authors surveyed patients’ preferences on two treatment choices; “one offering a modest length of survival, and the other offering a 50% chance of a substantially longer survival, but also a 50% chance of no additional survival” (31).

If patients care about long-term survival prospects, not just average survival, this study suggests the need to incorporate the valuation of risk preferences as a unique consideration in the HTA. Arguably, a two-step approach could be considered (40). First-step may comprise a standard cost-effectiveness assessment based on average clinical survival and other health benefits (i.e., QALY) estimates. Second-step may include other value considerations, both qualitatively and quantitatively (40). However, this can be achieved based on the assumption that patients acknowledge and can act on their own (risk) preferences. All in all, Lakdawalla et al. highlighted the view that value should incorporate the “perspective of the patient”, and value assessment frameworks should take this perspective into account.

Lakdawalla and Phelps (10), examined additional value elements, including ‘hope’, using a “generalized risk-adjusted cost-effectiveness (GRACE) model, which assumes that patients are utility maximizers in their choices about their treatment choices” (9, 10, 41). Building on the study by Garber and Phelps (37) and Lakdawalla et al. (41), this study authors incorporated patients’ risk preferences in treatment choices. Patients with “severe impairments and prospects of continued poor health, or those facing shorter life expectancy, were shown to have a higher willingness to pay for durable health gains” (41). Some patients may be willing to take the risk for a treatment that has a likelihood of ‘cure’ or ‘hope’ (31). The GRACE model suggested that “patients are not indifferent to the length of life or quality of life, which could indicate that marginal utility does not equal average utility” (41). The study authors indicated that “willingness to pay per QALY thresholds may need adjustments for value assessments” (41). However, it should be noted that this finding may have important implications on the ‘incentives’ for future innovation developers and investments (38).

Another method to quantify ‘hope’ was presented by using a discrete choice experiment (DCE) (42). Using a DCE for 200 patients with cancer or a history of cancer, Reed et al. (42), reported that “patients valued treatments with 5% and 10% chances of 10-year survival, independent of expected survival, although the findings did not hold in all scenarios” (42). First a pilot DCE was designed, and “participants were asked to assume that they had recently been diagnosed with cancer that had begun to metastasize” (42). Participants then had to consider choices “when expected survival was three years, with a given chance of 10-year survival, or a case with certain 3-year survival outcome (i.e., 10-year survival was zero)” (42). After piloting the DCE using general participants, cancer patients were asked to complete the online DCE survey. The study authors found that the “estimated value of ‘hope’ for a 5% chance of survival was on average about $6,000, and 10% for $12,500” (42). “With a life expectancy of 5 years, when a 20% chance of 10-year survival corresponds to 80% of an average 3.8 years, participants’ choices were consistent with expectations according to utility theory and risk neutrality” (42). However, when the choices had a “scenario with a life expectancy of 2 years, where a 20% chance of 10-year survival implies an 80% chance of a 1-month survival, patients rarely chose this option” (42). The study authors highlighted that there was heterogeneity in patients’ preferences across attributes. For example; “a latent class analysis, designed to identify groups with similar preferences, found four distinct groups of participants differing in terms of their sensitivity to costs and preferences for treatments enabling durable survival” (42). All in all, Reed et al. highlighted that the quantification of ‘hope’ is important, however, there are uncertainties about how much ‘hope’ may be worth and how to quantify heterogeneous preferences. Reed et al. concluded that researchers and policymakers should assess heterogeneous patterns of risk preferences and carefully consider them for resource allocation and reimbursement decisions (42).

All in all, these studies highlighted in Section 3.3 do not specifically focus on immunotherapy or NSCLC. Given the unique characteristics of immunotherapy, such as the “tail of the curve survival potential”, future studies that will present methods to quantify “hope” specifically for the valuation of ICIs are encouraged.

## 4 Discussion

Immunotherapy offers a distinctive mechanism of action compared to traditional treatments, arising from additional value dimensions that may not be captured in standard HTA methods. In this study, we focused specifically on the patients’ risk-seeking preferences because some cancer patients may prefer treatments that have a likelihood of durable survival. Based on the available evidence, our review revealed that economic value assessment methods should not only be based on survival improvement for the mean or median patient, but also on the quantification of risk preferences for durable overall survival gains. Although ICIs have distinctive characteristics that may increase the relevance of considerations of additional value dimensions, a major issue that should be considered against the inclusion of ‘hope’ relates to equity concerns. Higher spending on certain ICIs or other durable medical technologies that get extra importance based on ‘hope’ or patients’ stated preferences may have consequences (i.e., opportunity costs) inside and/or outside the health systems.

Our study showed that the quantitative methods that aim to measure ‘hope’ empirically are emerging. There are viewpoints that support and refute the inclusion of ‘hope’ alongside standard measures of health gain (7). The evidence on the quantification methods for ‘hope’ based on patients’ stated preferences for uncertain treatments is not strong and future research could strengthen this evidence base. One complexity is that the value in having access to therapies with different clinical profiles can diverge across patients, and across indications. Therefore, any attempt to add an empirical weighting for ‘hope’ at the population level may be premature.

In the United Kingdom, NICE includes additional criteria in the assessment of health technologies, in the end-of-life context as a modifier when considering its cost-effectiveness threshold (43). The intention is not to raise the threshold per se, but to give greater weight to QALYs achieved at the end-of-life (under certain circumstances), by focusing on the expected gain, rather than any element of ‘hope’. The International Society for Health Economics and Outcomes Research (ISPOR) Task Force researchers, who studied the US Value Assessment Frameworks, identified eight elements of value, and suggested considering additional value dimensions, including ‘hope’ (11). This ISPOR Task Force report recognizes, however, as did the recommendations of the Second Panel on Cost Effectiveness, that these additional value dimensions are subject to further research, and the methods for empirically integrating them into value frameworks are not yet validated (44, 45). There are also intrinsic equity concerns about “incorporating additional dimensions of value, without considering the opportunity costs and potential health losses that might be foregone”. Nonetheless, in its value assessment framework covering the years 2020 to 2023, ICER stated that it would consider broader value elements such as option value, the value of hope, and scientific spillovers in the ‘*other benefits or disadvantages*’ and ‘*contextual considerations*’ sections of ICER evidence reports (14). However, such considerations do not influence cost-effectiveness results or value-based prices.

Others have stated that “value elements, such as hope and fear, could legitimately affect individual decision-making but are fraught with difficulties in measurement as they relate to subjective experience and could be manipulated by the context” (39). Arguably, standard value assessments using the QALY also face measurement challenges, because the utility measurement can be a reflection of subjective experiences (38). Value elements such as ‘hope’ has the potential to be measured quantitatively using skewness in the outcome distribution (38). Improved measurement methods of value elements might allow modifiers to be applied quantitatively in the future, although challenges remain (38).

All in all, our review suggests the need for further research to develop and validate reliable methods for the quantification of ‘hope’ and valuation of risk preferences for patients treated with immunotherapy in NSCLC. The proliferation of empirical studies is promising, however, additional methods of development efforts are needed. If ‘hope’ were to be incorporated into the valuation of ICIs, the HTA evaluation would then need to depart from the traditional focus on average outcomes and include the notion that (some) patients may care about the distribution of durable benefits, not just the average.

## Author contributions

RZ: Conceptualization, data curation, writing – original draft, writing – review & editing. KR: Writing – review & editing. CU Writing – review & editing. All authors contributed to the article and approved the submitted version.
