# Supplementary material for: Incorporating risk preferences of patients in the valuation of immune checkpoint inhibitors for non-small cell lung cancer
Source: Front Oncol. 2023 Mar 8;13:1027659. doi: 10.3389/fonc.2023.1027659 (PMC10032401; doi:10.3389/fonc.2023.1027659)
Supplement: Supplementary file 1 [file Table_1.docx]

**Supplementary Table 1:** Search Syntax

| Category | Search terms |
| --- | --- |
| Population | “non-small cell cancer” AND (advanced OR metastatic OR recurrent) AND (“PD-1” OR “PD-L1” OR “CTLA-4” OR “programmed cell death” OR “cytotoxic T lymphocytes” OR “nivolumab OR ipilimumab” OR “immunotherapy” OR “immune checkpoint inhibitor”) |
| Intervention | AND (risk or stated) AND (preferences) OR (hope) |
| Comparison | N/A (no restriction) |
| Outcome | AND (quantitative OR methods OR methodology OR "value assessment" OR "value frameworks" OR "patient preferences" OR "value of hope") |
